# Author Correction: Action video game play facilitates “learning to learn”

**DOI:** 10.1038/s42003-021-02913-5

**Published:** 2021-12-07

**Authors:** Ru-Yuan Zhang, Adrien Chopin, Kengo Shibata, Zhong-Lin Lu, Susanne M. Jaeggi, Martin Buschkuehl, C. Shawn Green, Daphne Bavelier

**Affiliations:** 1 Institute of Psychology and Behavioral Science, Shanghai Jiao Tong University, 200030 Shanghai, China; 2 Shanghai Mental Health Center, Shanghai Jiao Tong University School of Medicine, 200030 Shanghai, China; 3 Department of Brain and Cognitive Sciences and Center for Visual Sciences, University of Rochester, Rochester, NY 14628 USA; 4 Faculté de Psychologie et Science de l’Éducation, University of Geneva, Geneva, Switzerland; 5 Campus Biotech, Geneva, Switzerland; 6 Sorbonne Université, INSERM, CNRS, Institut de la Vision, Paris, France; 7 Division of Arts and Sciences, NYU Shanghai, Shanghai, China; 8 Center for Neural Science and Department of Psychology, New York University, New York, NY 10003 USA; 9 NYU-ECNU Institute of Brain and Cognitive Science at NYU Shanghai, Shanghai, China; 10 School of Education and School of Social Sciences (Department of Cognitive Sciences), University of California, Irvine, Irvine, CA 92697 USA; 11 MIND Research Institute, Irvine, CA 92617 USA; 12 Department of Psychology, University of Wisconsin-Madison, Madison, WI 53706 USA

**Keywords:** Perception, Attention, Cognitive control, Human behaviour

Correction to: *Communications Biology* 10.1038/s42003-021-02652-7, published online 14 October 2021.

The original version of this Article contained funding information in the Acknowledgements section that was incorrectly given as ‘Swiss National Foundation Grant 100014_140676 (DB)’ and should have read ‘SwissNational Foundation Grant 100014_178814 (DB)’. This has now been corrected in both the PDF and HTML versions of the Article.

