# Supplementary material for: Action video game play facilitates “learning to learn”
Source: Commun Biol. 2021 Oct 14;4:1154. doi: 10.1038/s42003-021-02652-7 (PMC8517021; doi:10.1038/s42003-021-02652-7)
Supplement: Supplementary file 2 — Supplementary Information [file 42003_2021_2652_MOESM2_ESM.pdf]

SUPPLEMENTARY INFORMATION for

Action video game play facilitates “learning to learn”

Ru-Yuan Zhang, Adrien Chopin, Kengo Shibata, Zhong-Lin Lu, Susanne M. Jaeggi, Martin Buschkuehl, C. Shawn Green & Daphne Bavelier

Table of Contents

Supplementary Note 1: Procedures of the Cross-Sectional Study on Working Memory Learning

Supplementary Note 2: Statistical Analyses and Results of the Initial Intervention Study

Supplementary Note 3: Statistical Analyses and Results of the Replication Intervention Study

Supplementary Note 4: Statistical Analyses and Results of the Cross-Sectional Study on Working Memory Learning

Figure S1. Results of the Cross-Sectional Study on Working Memory Learning

Figure S2. Paradigm and Results of the Baseline Motion Learning in both the Initial Intervention Study and the Replication Intervention Study.

Supplementary Note 1: Procedures of the Cross-Sectional Study on Working Memory Learning Task.

Participants. Twenty non-video-game players and twenty habitual video game players (with 9 action video game players and 11 real-time strategy players) were recruited from the University of Maryland student body, under a protocol approved by the Institutional Review Board. The data from the 11-real-time strategy players were not considered in the analyses for the purposes of the present paper. All participants had normal or corrected-to-normal vision and provided informed written consent. Participants completed the video game playing questionnaire developed by the Bavelier laboratory[1]. We recruited non-video game players (NVGPs) as those who had less than 1 hour of action or of real time strategy game play per week in the past year as well as in the years before the past year. NVGPs were also required to have played less than 3 hours of video games per week of all other genres combined in all past years. We recruited action video game players (AVGPs) as those who played more than 5 hours of first- or third-person shooter games per week in the past year, or 3-5 hours per week of first- or third-person shooter games in the past year and more than 5 hours per week of first- or third-person shooter games in the years before the past year. In addition, AVGPs were required to have played less than 3 hours per week for any other video game genre in all past years. All participants were overtly recruited using campus-wide flyers.

Apparatus. The working memory learning task was programmed in E-prime 1.2 and presented on a Dell 19-inch LCD monitor.

Procedures. In the cross-sectional study, we used the same working memory learning task as above. Here, the whole procedure consisted of 10 sessions over 5 days, with two sessions per day separated by a 15-minute break in which participants were asked to take a short walk outside.

Supplementary Note 2: Statistical Analyses and Results of the Initial Intervention Study

Pre-test performance. We conducted a series of analyses to confirm that the two groups performances were matched before training. These are reported in the main text (statistical results) and Figure S2.

Effects of training on learning tasks at post-test. The results of the orientation learning and of the working memory learning tasks are reported in the main text.

Attentional Control Task - Multiple Object Tracking (MOT). At pretest, the participants' mean accuracy across the six difficulty levels of the attentional control task was computed and entered in a t-test. Results revealed comparable performance in the two groups ($t(23) = 1.69$, $p = 0.105$, Hedge's $g = 0.657$, $BF_{01} = 0.983$).

A 2 x 2 ANOVA with training group (action/control) and testing time (pre/post-test) was carried on participants' mean accuracy across the six difficulty levels of the attentional control task. This analysis revealed only a significant main effect of group (training group, $F(1, 23) = 5.35$, $p = 0.03$, partial $\eta 2 = 0.19$; testing time, $F(1, 23) = 0.673$, $p = 0.42$, partial $\eta 2 = 0.028$), indicating overall better performance in the action-trained group. The interaction between training group and testing time was not statistically significant ($F(1, 23) = 0.99$, $p = 0.33$, partial $\eta 2 = 0.041$). Yet, based on past results, we had expected an interaction between training group and testing time. A t-test indicated that the action group outperformed the control group at post-test albeit weakly ($t(23) = 2.46$, $p = 0.022$, Hedge's $g = 0.959$, $BF_{01} = 0.342$).

Gaming performance. The 45 hours of training were completed within a comparable time frame for both action and control trainees (action: 79.8 ± 6.2 days; control: 73.4 ± 7.0 days; two sample t-test, $p = 0.5$).

The game ability improvement on each game was evaluated by a paired t-test comparing game ability assessments conducted before and after training on that game. Action-trainees went through more checkpoints in all three assigned action games after training as compared to before training (Call Of Duty 1, $t(13) = 12.530$, $p < 0.001$, Hedge's $g = 3.04$, $BF_{01} < 0.00001$; Call Of Duty 2, $t(13) = 7.60$, $p < 0.001$, Hedge's $g = 2.29$, $BF_{01} = 0.0002$; Half-life 2, $t(13) = 5.57$, $p < 0.001$, Hedge's $g = 1.27$, $BF_{01} = 0.03$). Control trainees achieved more "life challenges" after training than before training (The Sims 3, $t(10) = 10.49$, $p < 0.001$, Hedge's $g = 2.90$, $BF_{01} < 0.0001$), purchased and attracted more animals in Zoo Tycoon ($t(10) = 6.73$, $p < 0.001$, Hedge's $g = 2.07$, $BF_{01} = 0.002$) and in Viva Piñata ($t(10) = 5.67$, $p < 0.001$, Hedge's $g = 1.95$, $BF_{01} = 0.006$). Thus, both groups showed significant improvements, as expected, after each 15-hour of game training.

Debriefing expectation questionnaire. This questionnaire probed the participants' belief on the purposes of the pre- and post-test tasks, how these tasks may relate to each other, and how game training may have changed performance on the tasks. Here we only report on this last aspect that probes participants' expectations about their training.

For the three pre-test tasks (attentional control task, baseline N-back task, and baseline motion learning task), a question was included probing participants' expectations prior to game training; we refer below to these as the type 1 questions:

Type 1. Think back to when you started the study. At the moment you were first assigned your video game to train on, which of the following answers below is closest to what you thought about how playing the game would change your ability if you were asked to perform this task again after playing the game:

a) I thought playing my assigned training video game would improve performance on this task
b) I thought playing my assigned training video game would not change my performance on this task
c) I thought playing my assigned training video game would worsen my performance on this task

d) I had no such thoughts
e) I do not remember

For all pre- and post-test tasks, the questionnaire included another question probing participants expectations at post-test (type 2 questions):

Type 2. Now that you have completed the study, which of the following answers below is closest to what you think now about how playing the game changed your ability to perform this task:

a) I think I performed better on this task because of my game training
b) I do not think my game training affected my performance on this task
c) I think I performed worse on this task because of my game training
d) None of the above

For both type 1 and type 2 questions, if participants answered, “I think I performed better on this task because of my game training”, their answer was assigned a response 1, and a response 0 otherwise. We carried out a Fisher’s exact test for type I and type II expectations.

For the attentional control task and the baseline N-back task, which participants completed both before and after training, we also explicitly asked all participants to rate their subjective belief regarding whether their performance on the given task improved from pre- to post-test using the following phrasing: Type 3. Finally how do you think your performance changed on this task from pre- to post-test (0 no change, -5 got much worse, +5 got much better)?

-5 -4 -3 -2 -1 0 1 2 3 4 5

We performed a simple two-sample t-test to contrast these ratings between the action and the control video game groups.

The groups’ expectations did not differ on the baseline motion learning task (Type 1, $p = 0.111$, $\varphi = 0.368$; Type 2, $p = 0.689$, $\varphi = 0.136$) administered at pre-test, nor on the working memory learning (Type 2, $p = 0.21$, $\varphi = 0.329$) and the orientation learning tasks (Type 2, $p = 0.24$, $\varphi = 0.277$) administered at post-test.

Participants in the action trained group were more likely to believe they performed better on the attentional control task because of their training than those in the control group (Type 1, $p = 0.005$, $\varphi = 0.592$; Type 2, $p = 0.005$, $\varphi = 0.592$). However, when asked about their performance

change from pre-to post-test (rather than just how well they did at post-test), no significant group difference was observed (Type 3, $t(23) = 0.59$, $p = 0.56$, Hedge’s $g = 0.248$, $BF_{01} = 2.381$. Furthermore, participants’ self-ratings of their pre- to post-test improvements on attentional control did not correlate with their true pre- to post-test performance change on attentional control as measured by mean MOT performance gain ($r = -0.134$, $p = 0.52$).

Regarding the baseline N-back task, the two training groups had similar expectations about performance at post-test (Type 1, $p = 0.18$, $\varphi = 0.309$; Type 2, $p = 0.230$, $\varphi = 0.327$). Concerning changes in performance from pre to post-test, no expectation difference was detected (Type 3, $t(23) = 0.094$, $p = 0.926$, Hedge’s $g = 0.04$, $BF_{01} = 2.696$) between the two training groups. Although some covariations existed between the self-rating change scores and participants’ actual improvement in the task, these correlations were all negative in both the action ($r = -0.581$, $p = 0.029$) and the control group (averaged improvement, $r = -0.567$, $p = 0.069$). Possible interpretations of these negative correlations remain unclear, but this clearly rules out expectation about improvement on the baseline N-back task as the source of the superior learning performance in the action group after game training.

Together, these results indicate action-trained participants expect to progress more than control-trained ones on the attentional control task; yet beliefs about possible change in performance, whether for the attentional control task or the baseline N-back task do not relate meaningfully to participants’ actual change in performance. Moreover, expectations about task performance at post-test were matched across training groups for all tasks, including the two key learning tasks. In all, these results provide little to no support for the view that participants’ expectations may mediate the impact of video game training on performance.

Age and gender. We wonder whether age and gender had an effect on learning parameters and no effect was observed. No difference existed between male and female neither in the orientation learning task (learning rate, $t(23) = -0.07$, $p = 0.94$, Hedge's $g = 0.03$, $BF_{01} = 4$; final performance, $t(23) = -0.36$, $p = 0.72$, Hedge's $g = -0.15$, $BF_{01} = 3.84$; initial performance, $t(23) = 0.33$, $p = 0.74$, Hedge's $g = 0.13$, $BF_{01} = 3.84$), nor in the working memory learning task (learning rate, $t(23) =$

1.32, p = 0.20, Hedge's g = 0.53, BF_{01} = 1.92; initial performance, t(23) = 0.29, p = 0.77, Hedge's g = 0.12, BF_{01} = 3.84). Age was not correlated with learning rates neither in the orientation learning task (r = -0.14, p = 0.49) nor in the working memory learning task (r = -0.21, p = 0.31).

Supplementary Note 3: Statistical Analyses and Results of the Replication Intervention Study

Pre-test performance. We report the results of the baseline motion learning task and the baseline N-back task in the main text.

Effects of training on learning tasks at post-test. We report the results of the orientation learning task and of the working memory learning task in the main text.

Pre-Post-Training ANOVA for perceptual learning tasks. The pre-registered analysis plan involved a mixed design ANOVA, with individuals' learning rate as the dependent variable, testing time (pre- vs. post-test) as a within-subject factor and training group (experimental vs. control) as a between-subject factor. Such an analysis is predicated on the idea that, although participants were not performing the same exact task at pre-test and post-test, these were nonetheless measures of the same fundamental ability. If this were the case, then performance at pre-test should be correlated to a reasonable degree with performance at post-test. Such a correlation though was not observed in either the initial intervention study or the replication study, as indicated by Spearman correlations between the learning rate parameters of the orientation and baseline motion learning tasks (initial intervention study, experimental group: r = -0.024 p = 0.94; control group: r = 0.1, p = 0.776; replication study, experimental group: r = 0.083 p = 0.68; control group: r = 0.077 p = 0.719). This suggests that a 2(group) x 2(testing time) ANOVA is not the correct analysis for demonstrating an effect of the training group on learning dynamics (in essence, the post-test is not a repeated measure of the pre-test). We thus decided to directly compare the model parameters between training groups at post-test - an analysis that we reported in the main text. However, for completeness with the pre-registered plan, we nonetheless report the 2x2 ANOVAs here.

We conducted the mixed-design ANOVA with individuals' learning rate as the dependent variable, testing time (pre- vs. post-test) as a within-subject factor and training group (experimental vs. control) as a between-subject factor. In this analysis, the key term is the interaction between training group and time. This interaction term approached, but did not reach significance (although in the direction stated in the pre-registration: $F(1,49) = 2.455$, $p = 0.124$, partial $\eta 2 = 0.048$). No main effect was significant (training group: $F(1,49) = 1.632$, $p = 0.207$, partial $\eta 2 = 0.032$; testing time: $F(1,49) = 0.177$, partial $\eta 2 = 0.004$, $p = 0.676$). Note that one control group participant who had missing baseline motion learning data was excluded from this analysis, but was included in the main text analysis.

Attentional Control Task - Multiple Object Tracking (MOT). Mean accuracy across the six difficulty levels of the attentional control task were averaged. We performed a t-test to compare participants' mean accuracy across groups, and found a small but significant difference between the two groups at pre-test ($t(50) = 2.1$, $p = 0.041$, Hedge's $g = 0.579$, $BF_{01}= 0.608$).

We also performed a 2 x 2 ANOVA with group (action/control) and testing time (pre/post-test) was carried on participants' mean accuracy across the six difficulty levels of the attentional control task. The main effect of group ($F(1, 50) = 3.86$, $p = 0.055$, partial $\eta 2 = 0.056$) was marginally significant, indicating a general better performance in the action group. As expected, the main effect of testing time ($F(1, 50) = 8.43$, $p = 0.005$, partial $\eta 2 = 0.14$) was detected, indicating a general training-induced enhancement of attentional control in both groups. We did not observe a significant interaction of group and time ($F(1, 50) = 0.05$, $p = 0.825$, partial $\eta 2 = 0.001$). A t-test revealed no significant group difference on the attentional control performance at post-test ($t(50) = 1.494$, $p = 0.141$, Hedge's $g = 0.408$, $BF_{01}= 1.442$).

Finally, in our pre-registration, we had posited a correlation between learning rate and attentional control at post-test. We further specified that this correlation would be seen in the action training group. In contrast to this prediction, we found no significant correlations between the performances on the attentional control task and orientation learning rates (action video game group: $r = 0.01$; $p = 0.952$) nor in the working memory learning task (action video game: $r = 0.29$;

p = 0.143). In the control group, a correlation existed between performance on the attention control task and orientation learning rate (r = 0.45, p = 0.023) but not working memory learning rate (r = 0.30, p = 0.14).

Gaming performance. The 45 hours of training were completed within a comparable time frame for both action and control trainees (action: 66.7 ± 3.6 days; control: 66.9 ± 6 days; two sample t-test, t(50) = 0.208, p = 0.836, Hedge’s g = 0.058, BF_{01} = 5.56).

In order to confirm that video game training was actively conducted at the participants home, we measured game ability pre- and post-training of all games (Action (n = 27): Half Life 2, Call of Duty: BlackOps 1, and Call of Duty: BlackOps 2, Control (n = 25): Sims 4, Zoo Tycoon 2013, and Viva Piñata). We evaluated the gaming improvements using quantifiable measures of gameplay collected every 15 hours of at-home-gaming in a 30-min session at the laboratory. The measures were the number of checkpoints attained in action video games and the number of completed challenges in controlled social video games. Distributions of checkpoint attained were normal except for the pre-training measurement of Call of Duty (Kolmogorov-Smirnov tests: KS = 0.36, p = 0.0013) and the post-training measurement of Viva Piñata (Kolmogorov-Smirnov tests: KS = 0.33, p = 0.0055), for which we used non-parametric statistics. The number of checkpoints attained in each game by the action video game group increased significantly with the training (Call of Duty 1, Wilcoxon test W = 325, p < 0.0001, Hedge's g = 1.24, BF_{01} = 0.0084; Call of Duty 2, paired t-test t(26) = 4.73, p < 0.0001, Hedge’s g = 0.9, BF_{01} = 0.91; Half-life 2, t(26) = 7.1, p < 0.0001, Hedge’s g = 1.37, BF_{01} < 0.0001). The number of completed challenges in the control video games also increased significantly (paired t-tests: The Sims, t(24) = 7.04, p < 0.0001, Hedge’s g = 1.4, BF_{01} = 0.00056).; Zoo tycoon, t(24) = 5.46, p = 0.00001, Hedge’s g = 1.1, BF_{01} < 0.0001; Viva Piñata, W = 208, p < 0.0001, Hedge’s g = 0.51, BF_{01} = 0.30). Therefore, both groups showed significant improvements in the respective games after game training.

Expectation questionnaire. We assessed participant’s expectations using a questionnaire based on ref. [2]. This questionnaire tested expectations on how different intervention regimes may affect

participants in one of the four following domains: cognition, mood, productivity at work, and physical fitness. Note that our only interest in this case was with respect to the cognitive domain. Other domains were probed primarily to make it more difficult for participants to surmise which was our main interest. Each domain was evaluated with 2 similar questions. For example, the two questions for cognition are:

1) I expect playing such video games will enhance my cognition.

2) I expect that after playing such video games my cognition will be considerably better.

The scales include 4 response items (*partially disagree, partially agree, agree, definitively agree*), and we averaged the scores of the two questions. There were group differences in the cognitive domain (t-test, $t(50) = 3.27$, $p = 0.002$, Hedge's $g = 0.91$, $BF_{01} = 0.06$).

Notably, these expectations in the cognition domain correlated neither with actual learning rates in the orientation learning task (action video games: $r = 0.08$, $p = 0.68$; control video games: $r = -0.04$, $p = 0.85$) nor with the actual learning rates in the working memory learning task (action video games: $r = -0.32$, $p = 0.11$; control video games: $r = -0.06$, $p = 0.77$).

Thus, while participants in the action video game group reported that they expected to perform better in the cognitive domain than participants in the control video game group, their participants' expectations did not predict their actual behavior.

IMI and Flow score - correlation with learning rate

<u>IMI Questionnaire:</u> The intrinsic motivation questionnaire assesses the extrinsic and intrinsic reward dimensions[3]. We administered the questionnaire to assess the motivation related to each assigned game. Twenty-five questions with six dimensions were administered on a 7-point Likert scale ranging from *not at all true* (1) to *very true* (7). We averaged the 1-7 scores of all the items in each subscale (after inverting the scores of the items needing inversion). We also averaged the three measures of IMI (at times +15h, +30h and +45h of training). The intrinsic motivation score did not correlate with learning rates of the orientation learning task (action video games: $r = 0.08$, $p = 0.676$; control video games: $r = -0.10$, $p = 0.63$) nor the working memory learning task (action video games: $r = -0.08$, $p = 0.70$; control video games: $r = 0.07$, $p = 0.75$).

Flow State Scale Questionnaire: The questionnaire measures the positive mental state that occurs when a participant is fully engaged in the game and personal skills typically equal the required challenge [4]. The questionnaire consists of 36 questions with nine dimensions, answered on a 5-point Likert scale ranging from *strongly disagree* (1) to *strongly agree* (5). We averaged the 1-5 scores of all questions. We also averaged together the three measures of flow state (at times +15h, +30h and +45h of training). Flow states did not correlate with the learning rates of the orientation learning task (action video game group: $r = -0.02$, $p = 0.92$; control video game group: $r = -0.05$, $p = 0.81$) nor the working memory learning task (action video game group: $r = -0.24$, $p = 0.22$; control video game group: $r = -0.13$, $p = 0.52$).

Age and gender. We wonder whether age and gender had an effect on learning parameters and no effect was observed. No difference existed between male and female neither in the orientation learning task (learning rate, $t(50) = 1.07$, $p = 0.29$, Hedge's $g = 0.3$, $BF_{01} = 3.22$; final performance, $t(50) = -0.73$, $p = 0.47$, Hedge's $g = -0.21$, $BF_{01} = 4.34$; initial performance, $t(50) = -1.12$, $p = 0.26$, Hedge's $g = -0.32$, $BF_{01} = 3.12$), nor in the working memory learning task (learning rate, $t(50) = 0.97$, $p = 0.34$, Hedge's $g = 0.27$, $BF_{01} = 3.57$; initial performance, $t(50) = 1.11$, $p = 0.27$, Hedge's $g = 0.31$, $BF_{01} = 3.12$). Age was not correlated with learning rates neither in the orientation learning task ($r = 0.01$, $p = 0.96$) nor in the working memory learning task ($r = -0.09$, $p = 0.52$).

Supplementary Note 4: Statistical Analysis and Results of the Cross-Sectional study on Working Memory Learning

We performed the hierarchical Bayesian analyses separately on each group. Consistent with the "learning to learn" hypothesis, a significantly higher learning rate was seen in the action video game players (AVGPs) as compared to non-video game players (NVGPs) (Fig. S2, $t(27) = 12.33$, $p < 0.001$, Hedge's $g = 5.19$, $BF_{01} < 0.0001$). Meanwhile, initial performance (as measured by the intercept in our model) was not significantly different between the two groups ($t(27) = 1.02$, $p = 0.319$, Hedge's $g = 0.42$, $BF_{01} = 1.863$).

Figure S1

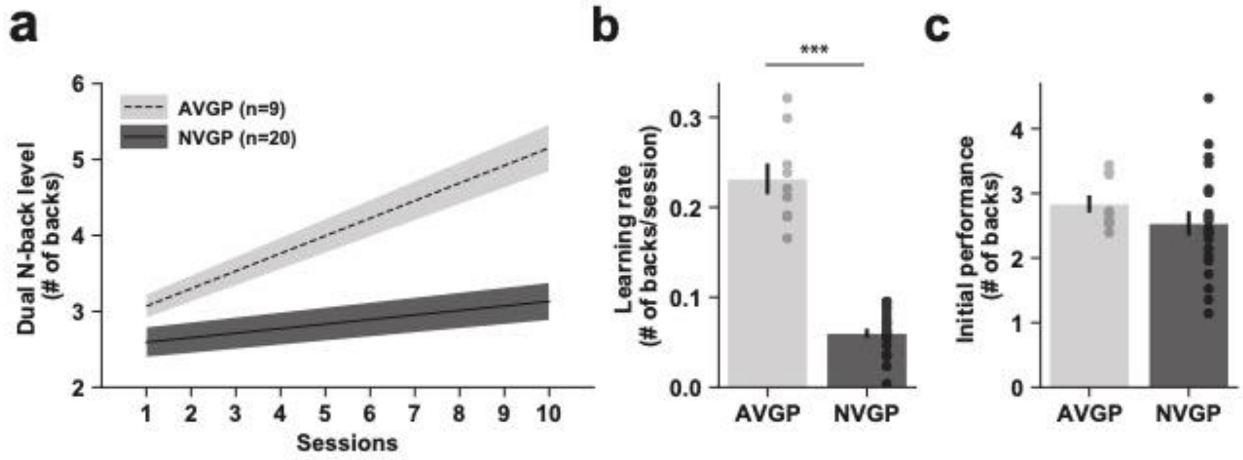

Cross-sectional study on working memory learning.

Panel A. Group learning curves of habitual action video game players (AVGP) and non-video-game players (NVGP). The upper and lower bounds of the shaded area are learning curves plotted using the values of the group mean +/- S.E.M. ***Panel B-C.*** Bayesian estimates of learning rate and initial performance of the two groups. The significantly higher learning rates in the AVGP group is consistent with the findings in the two intervention studies reported in the main text. All error bars are S.E.M. across participants. Black and gray circles correspond to each participant individual data.

Figure S2

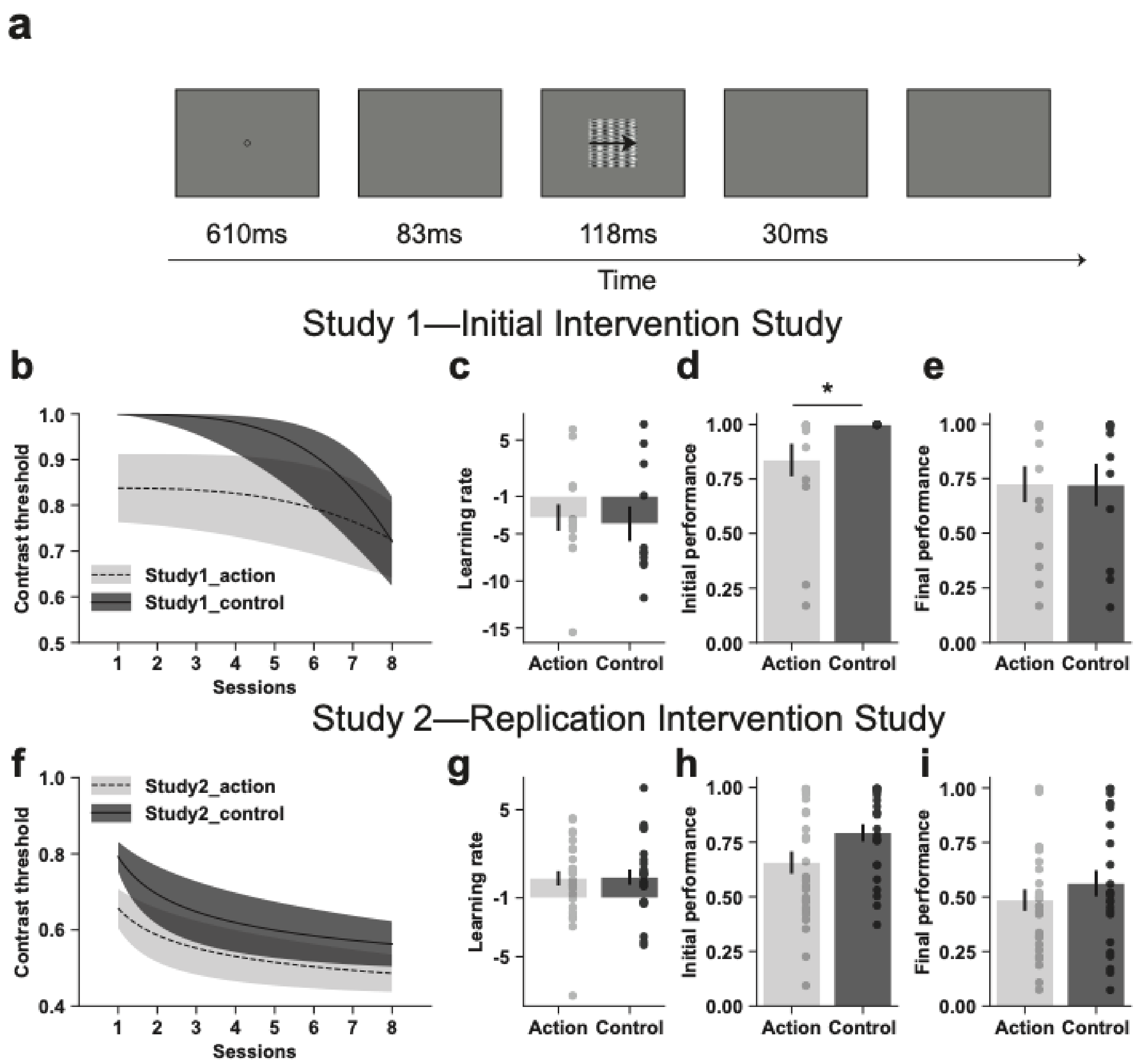

Comparable learning ability of the two groups in the baseline motion learning task.

Panel A illustrates the baseline motion learning task in both intervention studies. On each trial, participants view a grating drifting either to left or right and press a button to report the motion direction. Horizontal noise pixels are added to the grating stimuli to increase task difficulty. ***Panel B.*** Group level learning curves for the initial intervention study. The upper and lower bounds of

the shaded area are learning curves plotted using the values of the group mean +/- S.E.M. ***Panels C-E.*** learning rate, initial performance, and final performance estimated by hierarchical Bayesian modeling in the initial intervention study. The two groups had comparable characteristics of learning. ***Panels F-I.*** Similar to panels B-E but for the replication intervention study. All error bars are S.E.M. across participants. Black and gray circles correspond to each participant individual data.

Supplementary References

1 Green, C. S. *et al.* Improving methodological standards in behavioral interventions for cognitive enhancement. *Journal of Cognitive Enhancement*. **3**, 2-29 (2019).

2 Barth, J., Kern, A., Luthi, S. & Witt, C. M. Assessment of patients' expectations: development and validation of the Expectation for Treatment Scale (ETS). *BMJ Open*. **9**, e026712 (2019).

3 Kanfer, R. & Ackerman, P. Individual Differences in Work Motivation: Further Explorations of a Trait Framework. *Appl. Psychol.* **49**, 470-482 (2001).

4 Jackson, S. A. & Marsh, H. W. Development and Validation of a Scale to Measure Optimal Experience: The Flow State Scale. *Journal of Sport and Exercise Psychology*. **18**, 17-35 (1996).
