# Supplementary material for: Action video game play facilitates “learning to learn”
Source: Commun Biol. 2021 Oct 14;4:1154. doi: 10.1038/s42003-021-02652-7 (PMC8517021; doi:10.1038/s42003-021-02652-7)
Supplement: Supplementary file 3 — Reporting Summary [file 42003_2021_2652_MOESM3_ESM.pdf]

nature research

Corresponding author(s): Daphné Bavelier

Last updated by author(s): 2021/7/26

Reporting Summary

Nature Research wishes to improve the reproducibility of the work that we publish. This form provides structure for consistency and transparency in reporting. For further information on Nature Research policies, see our Editorial Policies and the Editorial Policy Checklist.

Statistics

For all statistical analyses, confirm that the following items are present in the figure legend, table legend, main text, or Methods section.

n/a	Confirmed	
☐	☒	The exact sample size (*n*) for each experimental group/condition, given as a discrete number and unit of measurement
☐	☒	A statement on whether measurements were taken from distinct samples or whether the same sample was measured repeatedly
☐	☒	The statistical test(s) used AND whether they are one- or two-sided<br>*Only common tests should be described solely by name; describe more complex techniques in the Methods section.*
☐	☒	A description of all covariates tested
☐	☒	A description of any assumptions or corrections, such as tests of normality and adjustment for multiple comparisons
☐	☒	A full description of the statistical parameters including central tendency (e.g. means) or other basic estimates (e.g. regression coefficient) AND variation (e.g. standard deviation) or associated estimates of uncertainty (e.g. confidence intervals)
☐	☒	For null hypothesis testing, the test statistic (e.g. F, t, r) with confidence intervals, effect sizes, degrees of freedom and P value noted<br>*Give P values as exact values whenever suitable.*
☐	☒	For Bayesian analysis, information on the choice of priors and Markov chain Monte Carlo settings
☐	☒	For hierarchical and complex designs, identification of the appropriate level for tests and full reporting of outcomes
☐	☒	Estimates of effect sizes (e.g. Cohen's d, Pearson's r), indicating how they were calculated

Our web collection on statistics for biologists contains articles on many of the points above.

Software and code

Policy information about availability of computer code

Data collection	All the tasks used in this manuscript have already been published and fully described in previous articles (referenced in our manuscripts). We used Matlab R2017a and the PsychToolBox 3 to code them.
Data analysis	Codes for analysis are provided here: https://osf.io/4xe59. Parts of the analyses were done using SPSS 12.0 and JASP 0.14.

For manuscripts utilizing custom algorithms or software that are central to the research but not yet described in published literature, software must be made available to editors and reviewers. We strongly encourage code deposition in a community repository (e.g. GitHub). See the Nature Research guidelines for submitting code & software for further information.

Data

Policy information about availability of data

All manuscripts must include a data availability statement. This statement should provide the following information, where applicable:

- Accession codes, unique identifiers, or web links for publicly available datasets
- A list of figures that have associated raw data
- A description of any restrictions on data availability

Data for analysis are provided here: https://osf.io/4xe59

Field-specific reporting

Behavioural & social sciences study design

All studies must disclose on these points even when the disclosure is negative.

Study description	Two randomized controlled intervention studies (quantitative) and one cross-sectional quantitative study (supplements)
Research sample	Study 1: 36 adult participants, younger than 35, recruited in Rochester, NY, USA. Participants had little to no experience with gaming.<br>Study 2: 64 adult participants, younger than 35, recruited in Geneva, Switzerland. Participants had limited experience with gaming.
Sampling strategy	For all studies, sampling happened by convenience.<br>Study 1: we sampled as many participants as possible in the time frame of one year (last year of Ph.D for RYZ).<br>Study 2: we pre-registered the procedure with a sample size estimating from the results of study 1, using a power analysis (https://osf.io/629yx). We sent invitations every month to 20 eligible participants, and stopped offers when 64 participants had completed the pre-test part of the study, which allowed for an attrition rate of approximately 20%.
Data collection	We describe data collection fully and in great details in the method section.
Timing	Study 1: sept 2014- dec 2015<br>Study 2: Jan 2018 - June 2018
Data exclusions	Study 1: Criteria for exclusion were decided in advance. One control trainee was excluded because of technical problems with the apparatus.<br>Study 2: Criteria for exclusion were pre-registred. Six participants were excluded before training as they demonstrated no learning in the motion discrimination task (pre-training estimated learning rate of 0), another 3 had to be excluded (1 because of age outside of decided limits, 1 because they were not naive to the conditions, and 1 because they failed to comply with the procedures of the study). One participant was removed from the analysis of the results in the baseline motion learning task because of a technical issue at the end of their first session; testing continued with session 2 but we could not interpolate the result of session 1.
Non-participation	Study 1: Six participants failed to comply with the at-home video game training protocol and one action trainee withdrew due to game-induced motion sickness (21% non-participation rate).<br>Study 2: Eight participants dropped out during the study (13.3% non-participation rate).
Randomization	Study 1: pseudo-random fashion so as to balance gender across training groups<br>Study 2: Training group assignment was randomized using the minimization method. We applied the Efron's biased coin technique separately for each of the 4 following strata combining age and gender: 18-26-years-old males, 27-35-years-old males, 18-26-years-old females, 27-35-years-old females.

Reporting for specific materials, systems and methods

We require information from authors about some types of materials, experimental systems and methods used in many studies. Here, indicate whether each material, system or method listed is relevant to your study. If you are not sure if a list item applies to your research, read the appropriate section before selecting a response.

Materials & experimental systems

n/a	Involved in the study	
x		Antibodies
x		Eukaryotic cell lines
x		Palaeontology and archaeology
x		Animals and other organisms
	x	Human research participants
x		Clinical data
x		Dual use research of concern

Methods

n/a	Involved in the study	
x		ChIP-seq
x		Flow cytometry
x		MRI-based neuroimaging

Human research participants

Policy information about studies involving human research participants

Population characteristics	Study 1: The final sample consisted of 14 participants in the action video game group (7 women; 18–34 years old, mean age 23 years) and 11 participants (9 women; 19–56 years old, mean age 24.3 years) in the control video game group.<br>Study 2: The final sample consisted of 27 participants in the action video game group (11 women; 19–35 years old, mean age 23 years) and 25 participants (11 women; 18–33 years old, mean age 22.8 years) in the control video game group.
Recruitment	Both studies recruited through flyers. Flyers mentioned playing video games, so that it could have biased recruitement toward participants that have positive attitudes for video games, and potentially positive expectations on how video games impact their cognition. Note that both arms of the study required playing video games; in addition, we tested for the effect of expectations in our analyses, and found similar expectations about how the assigned video game play may impact cognition in the experimental and control-trained group.
Ethics oversight	study 1: This study was run under a protocol approved by the University of Rochester Research Subjects Review Board.<br>study 2 :This study was run under a protocol approved by the University of Geneva Research Subjects Review Board.

Note that full information on the approval of the study protocol must also be provided in the manuscript.
